# The phylogenetics of *Leucocytozoon caulleryi* infecting broiler chickens in endemic areas in Indonesia

**DOI:** 10.14202/vetworld.2017.1324-1328

**Published:** 2017-11-11

**Authors:** Endang Suprihati, Wiwik Misaco Yuniarti

**Affiliations:** 1 Department of Parasitology, Faculty of Veterinary Medicine, Universitas Airlangga, Jl. Mulyorejo, Kampus C Unair, Surabaya, 60115, Indonesia; 2 Department of Clinical Science, Faculty of Veterinary Medicine, Universitas Airlangga, Jl. Mulyorejo, Kampus C Unair, Surabaya, 60115, Indonesia

**Keywords:** broiler chickens, cytochrome b gene, endemic, Indonesia, *Leucocytozoon caulleryi*

## Abstract

**Aim:**

The objective of this research was to determine the species and strains of *Leucocytozoon caulleryi* and study the phylogenetics of *L. caulleryi* of broiler chickens in endemic areas in Indonesia.

**Materials and Methods:**

Blood samples were collected from broiler chickens originated from endemic area in Indonesia, i.e., Pasuruan, Lamongan, Blitar, Lumajang, Boyolali, Purwokerto, and Banjarmasin in 2017. Collected blood was used for microscopic examination, sequencing using BLAST method to identify the nucleotide structure of cytochrome b (*cyt b*) gene that determines the species, and the phylogenetics analysis of *L. caulleryi* that infected broiler chickens in endemic areas in Indonesia, using Mega 5 software.

**Results:**

The results showed that Plasmodium sp. and *L. caulleryi* were infected broiler chickens in endemic areas in Indonesia. *L. caulleryi* in one area had very close phylogenetic relations with those in other areas. The genetic distance between *L. caulleryi* taxa from various endemic areas is very close (<5%).

**Conclusion:**

There is a very close phylogenetics among strains of *L. caulleryi* that infected broiler chickens in various endemic areas in Indonesia.

## Introduction

Leukocytozoonosis is a parasitic disease in poultry which is caused by protozoans from the *Leucocytozoon* genus. These protozoas live as parasites in the white blood cells. In Southeast Asia, there are two species that cause leukocytozoonosis in chickens the most, which are *Leucocytozoon caulleryi* and *Leucocytozoon sabrazesi*. This disease occurs very frequently in chicken farms that are close to water sources. This is because water sources are the natural habitat of *Leucocytozoon* sp. vectors, which are *Simulium* sp. and *Culicoides arakawae* [1].

In endemic areas, this disease occurs throughout the year. There is a positive correlation between leukocytozoonosis incidences with the season and farm locations. During seasonal changes, from rainy season to dry season and vice versa, the frequency of leukocytozoonosis incidences tends to increase. This is due to the increase in the populations of *Simulium* sp. and *Culicoides* sp. [2].

The outbreaks of leukocytozoonosis in broiler chickens in East Java are about 32% and in Central Java about 67%, while the incidences of leukocytozoonosis in local chickens and birds were not reported [3]. The morphological identification of *Leucocytozoon* sp. is often unable to be set up to the species determination due to morphological variations which make it hard to characterize the parasite’s morphology.

Molecular biology research can assist in species determination and can even show genetical varieties of *Leucocytozoon* between species [4,5]. Up until now, there has been little information on the genetical analyses of *Leucocytozoon* in chickens, compared to other protozoas. Even, there has not been information yet about the phylogenetic analysis that gives information about the varieties of *Leucocytozoon* that infect chickens in Indonesia up until now.

The genetic variations that create virulent strains in the microorganisms are thought to be responsible for poultry disease outbreaks. Phylogenetic analysis can be used as the basis for vaccine making for specific species [6,7].

Deoxyribonucleic acid (DNA) analysis on *Leucocytozoon* has been conducted to cytochrome b (*cyt b*) gene [8]. Series of genetic information in the mitochondrial DNA was reported to be able to portray the characteristics of a population and phylogenetics and able to reconstruct the evolutionary history [9,10]. *cyt b* gene is one of the genes located in the mitochondrial genome that is widely used for molecular analyses. *cyt b* gene possesses fast and slow developments of codon positions which have conservative parts and ever-changing parts [11].

## Materials and Methods

### Ethical approval

The experiments and sampling procedures were conducted under the protocol reviewed and approved by the Ethics Committee at Universitas Airlangga

### Sampling

**Figure-1:**
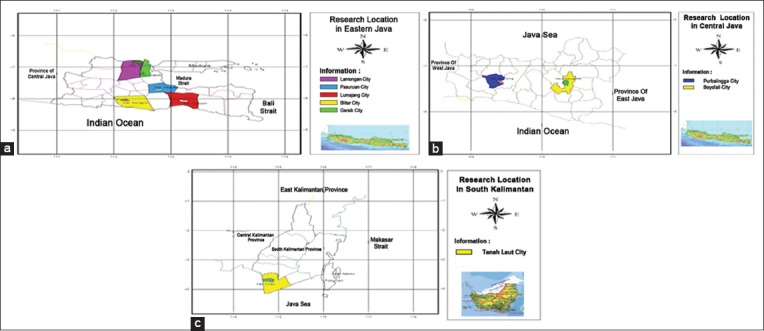
(a-c) The map of research sampling location, East Java, Central Java, and East Kalimantan.

All samples were taken from chicken originating from endemic areas in Indonesia in 2012. These areas include Pasuruan (n = 10), Lamongan (n = 6), Blitar (n = 10), Lumajang (n = 4), Boyolali (n = 10), Purwokerto (n = 8), and Banjarmasin (n = 15)(Figure-1).

Blood samples were collected and stored, and then, samples were fixed and stained for microscopic observation. The remainders of the blood samples were used for the subsequent DNA extraction procedure.

### Polymerase chain reaction (PCR) and sequencing

Blood samples were extracted using phenol-chloroform. Extracted DNA was dissolved in TE solution and used for nested PCR. The primer design was based on a pre-existing sequence, either from the previous research data or the data contained in GenBank. Two primers were used in the first- and second-round PCR. Nucleotide sequence for first round was 5’- CATATATTAAGAGAATTATGGAG-3’ and 5’-ATAAAATGGTAAGAAATACCATTC-3’ and for the second round was 5’- ATGTGCTTTAG ATATATGCATGCT-3’ and 5’-GCATTATCTGGATGTGATAATGGT-3’ [12,13].

In the first cycle, the total volume for this reaction was 25 μl reaction mixture consisted of 2 μl DNA template, 0.125 mM dNTP, 0.2 μM primer, 3 mM MgCl_2_, and 0.25 units Taq polymerase and buffer. For the primary PCR, a total of 35 cycles was carried out, consisting of denaturation at 94°C for 30 s, annealing at 50°C for 30 s, and extension at 72°C for 45 s. The second PCR reaction was performed in a total of 40 cycles identical to the first PCR, except the used of 0.4 μM primers, 0.5 units Taq Polymerase, and 0.2 μl of the first PCR product were used as the DNA template.

The amplification product was visualized in 2% agarose gels stained with ethidium bromide. The expected base length is 600 bp in the first round and 503 bp in the second round.

### Determining *cyt b* nucleotide gene sequence

This activity was carried out to know the variations of DNA sequence that was produced from the PCR sequencing with a DNA template originating from *Leucocytozoon* genome. The PCR sequencing process of *cyt b* gene used ABI PRISM 310 (Applied Biosystems, Foster City, CA, USA) automated sequencer.

The reaction was conducted by mixing the DNA template (40 μg), which was the result of amplification and purification, with 1 μL primer (reverse primer) at 2 pmol/μL and 4 μL concentrations of the ready mix (containing Ampli Tag and fluorescent-labeled dNTP). This mix was amplified in a PCR at a temperature of 96°C for 30 s, 50°C for 15 s, and 60°C for 4 min until 25 rounds. The PCR result was centrifuged at 12,000 rpm for 1 min, the liquid of which being discarded, and then centrifuged again at 14,000 rpm for 2 min for drying. The lower tube was then discarded and changed with a new Eppendorf tube.

Elution Buffer in the amount of 30 μL was added and centrifuged at 10,000 rpm for 1 min. In this process, a pure DNA had been produced. For the sequencing, a 4 μL Big Dye sequencing kit was mixed with a 4 μL DNA sequencing kit (such as Big Dye Terminator) and 5 μL of pure DNA. Electrophoresis was carried out with 4% of acrylamide gel in a TBE 1 × running buffer using ABI 310 DNA sequencer.

### Analysis of phylogenetic sequence of DNA Leucocytozoon

Molecular data preparation to build a phylogenetic tree using genetic distance approach was conducted with Mega 5 software [14]. A standard genetic distance was used to compare genetic distance in each of the population samples [15].

## Result

### Microscopic L. caulleryi identification

**Figure-2:**
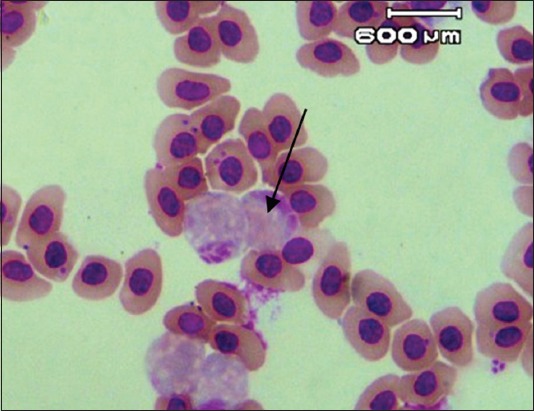
Gamet of *Leucocytozoon caulleryi* (Olympus CX-21 microscope with 1000× magnification; 2.0-megapixel OptiLab Digital Camera).

Blood smear with Giemsa staining and examination using PCR showed that *Leucocytozoon* found in this study was entirely *L. caulleryi* (Figure-2) [16].

### Determining Leucocytozoon species with BLAST method

The overall results of identification *L. caulleryi* with the BLAST method for the mtDNA *cyt b* gene of *Leucocytozoon* can be seen in Table-1. All found *Leucocytozoons* were detected as *L. caulleryi*, while samples from Lumajang and Blitar2 were detected as *Plasmodium juxtanucleare*. Plasmodium found in this research was detected with the first-round PCR. In general, *L. caulleryi* in those various areas has a very low genetic distance with *L. caulleryi* (AB302215).

**Table-1:** Identification of *Leucocytozoon caulleryi* with BLAST method in mtDNA *cyt b* gene PCR product of *Leucocytozoon.*

Location	*cyt b* gene 600 bp Round I	*cyt b* gene 503 bp Round II	BLAST results
Blitar	2	2	1+1*
Lamongan	2	2	2
Pasuruan	4	4	4
Lumajang	2	2	*
Boyolali	1	1	1
Purwokerto	2	2	2
Banjarmasin	2	2	2

* Identified as *Plasmodium*. PCR=Polymerase chain reaction, DNA: Deoxyribonucleic acid

### The alignment and phylogenetic tree of L. caulleryi in broiler chickens in endemic areas in Indonesia

**Figure-3:**
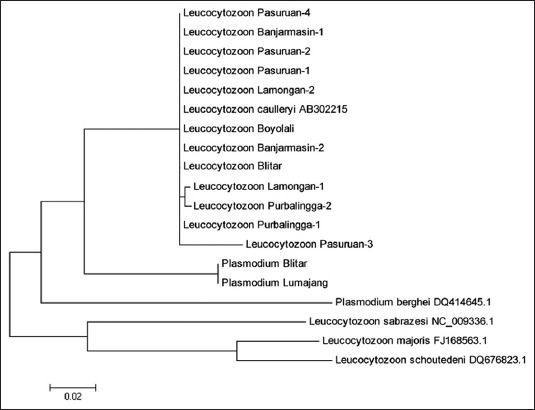
The phylogenetic tree of *cyt b* genes (503 bp) of *Leucocytozoon* caulleryi in broiler chickens in endemic areas in Indonesia, n=12 and *Plasmodium juxtanuclear* in broiler chickens in Blitar and Lumajang. *Plasmodium berghei* was retrieved from Genbank as an out group and *Leucocytozoon* sp. from other species was taken from GenBank.

From the alignment process, it was revealed that there was a slight difference, which, in general, showed that the genetic distance was very low (<5%). Among those taxa, it appeared that *L. caulleryi* from Pasuruan 3 had the biggest mutation, followed by Lamongan 1 and Purwokerto 2 (Figure-3).

However, if *L. caulleryi* strains from those areas was compared with other *Leucocytozoon* sp. and *Plasmodium* sp. (Figure-3), the *L. caulleryi* in this research was located in one clade which showed that they had a very close phylogeny.

It means that *L. caulleryi* was genetically closer to *Plasmodium* sp. than to other species of *Leucocytozoon*. The Plasmodium that was found in Blitar and Lumajang was *P. juxtanucleare* (BLAST checking), which its morphology was similar to *L. caulleryi*.

## Discussion

The finding of *Plasmodium* sp. whose morphology and PCR products resemble *L. caulleryi* could be explained ecologically due to the evolution of *L. caulleryi* in several endemic areas caused by the interference of humans who traded poultry [17].

Some isolates of *L. caulleryi* from different regions (Boyolali and Lamongan 2) have the same nucleotide sequence (0% genetic distance) but among them exhibit different morphologies. This may be due to changes in protein caused by environmental influences or stress that may alter a protein configuration called folding protein [18]. The low genetic distance can also be caused by the trading of infected chicken by *Leucocytozoon* between the cities.

Among the taxon, *L. caulleryi* from Pasuruan 3 has the most mutation, followed by Lamongan 1 and Purwokerto 2. Mutations can lead the changes of parasite characters, such as the pathogenicity and the variation of its life cycle. Therefore, its need a continuous observation of the case leukocytozoonosis in Pasuruan.

Pasuruan 3 isolate was not in the same group with isolates from Pasuruan 1 and Pasuruan 2 although they were originated from the same area. One of the causes was probably the same with the above explanation. Meanwhile, the spread of *L. caulleryi* was done by *Culicoides* sp. Therefore, there should be further studies to find the factors that caused the spread of *L. caulleryi* among the hosts and across areas. Even though *L. caulleryi* from Pasuruan 3 was outside the groups of other areas, the genetic distance was still < 5%.

The results of the research showed that the intraspecies genetic distance of *L. caulleryi* from various endemic areas with *L. caulleryi* (AB 302215) on average was < 5%. The genetic variety of intraspecies in this research were supported the occurrence of morphological variation. The less than 5% genetic distance in *cyt b* gene portrayed intraspecific species variation, while in *Leucocytozoon* parasite, this < 5% genetic distance showed interspecific species genetic variety. These data were in line with another study that stated that two parasite species in poultry, *Haemoproteus* sp. and *Plasmodium* sp., also with genetic distance of less than 5% for the *cyt b* gene, had different morphologies [12].

It is possible that the criteria of genetic distance of < 5% in *cyt b* gene show the interspecific species variety in many groups of hemosporidians that infect poultry, and thus, it can be used to explain the nature of the phylogenetic tree of this gene. It should also be noted that genetic diversity of the *cyt b* genes in some species of poultry parasite whose morphological characteristics have been identified indicates genetic distance < 5%, even some species have a genetic distance of < 1% [5]. The genetic distance criteria < 5% in the *cyt b* gene can be developed to identify species in the hemosporidia group cautiously. It is suggested to combine molecular data with microscopic one.

Data of the genetic distance between species that morphologically clear were very important. Increasing data in this field may underlie phylogenetic tree genetic of *cyt b* gene of poultry hemosporidia and help to build parasitic taxonomy based on molecular data [5].

The closeness among *L. caulleryi* species in various areas was analyzed with phylogenetic tree analysis that showed the close genetic relationship as the cause of leukocytozoonosis in broiler chickens, including with *L. caulleryi* from GenBank.

*L. caulleryi* from Pasuruan 3 showed the most distant genetic relationship, which was allegedly caused by a mutation in several points. This mutation may due to drug exposure or the existence of chickens brought from other areas to Pasuruan. The mutation had caused changes in the characteristics of this parasite, that is, the pathogenicity and its life cycles.

The spread of isolates which was gotten from a phylogenetic analysis requires further studies. The isolates from Banjarmasin 1 and Banjarmasin 2 (South Kalimantan), for example, belonged to the same group with isolates from Blitar, Pasuruan 1, and Pasuruan 2 (East Java). The structural similarities of *L. caulleryi* in these areas were due to various factors. One of which was probably caused by living animal transportation across regions.

The phylogenetic tree in Figure-2 describes the closeness between *L. caulleryi* and *Plasmodium* sp. from Blitar and Lumajang, whereas this parasite has similar morphology with *L. caulleryi*. The two *Plasmodium* sp. isolates were close to *L. caulleryi* and both were from the same strain. The *Plasmodium* sp. found in this research had a lower genetic distance with *L. caulleryi* than with other species of *Leucocytozoon*.

This is an indication that the morphological data of *Leucocytozoon* had to be supported with genetic data to reveal the level of varieties and to describe the taxonomy of *Leucocytozoon*.

The results of this research showed that there was a different molecular taxonomic concept with the previous concept, where *Plasmodium* sp. had a different genus with *Leucocytozoon* sp. Therefore, there should be a rearrangement of taxonomic concept that needs to adjust molecular data with morphological. *L*. *sabrazesi* also attacks chickens, even though the host is the same, but it has a very distant phylogeny with *L. caulleryi* (Figure-3).

## Conclusion

*Leucocytozoon* found in broiler chickens in endemic areas in Indonesia is *L. caulleryi*. The genetic distance between *L. caulleryi* taxa from various endemic areas is very close (<5%). There is a very close phylogenetics among strains of *L. caulleryi* that infected broiler chickens in various endemic areas.

## Authors’ Contributions

ES and WMY carried out the main research works, WMY performed the statistical analysis and analyzed the main data in the experiments, and ES and WMY approved the final manuscript. All authors read and approved the final manuscript.
